# The Effectiveness and Cost-effectiveness of Well Parent Japan for Japanese Mothers of Children With ADHD: Protocol for a Randomized Controlled Trial

**DOI:** 10.2196/32693

**Published:** 2022-04-19

**Authors:** Shizuka Shimabukuro, David Daley, Takahiro Endo, Satoshi Harada, Akemi Tomoda, Yushiro Yamashita, Takashi Oshio, Boliang Guo, Atsuko Ishii, Mio Izumi, Yukiko Nakahara, Kazushi Yamamoto, Akiko Yao, Gail Tripp

**Affiliations:** 1 Human Developmental Neurobiology Unit Okinawa Institute of Science and Technology Graduate University Onna-son, Okinawa Japan; 2 Division of Psychiatry and Applied Psychology School of Medicine University of Nottingham UK Nottingham United Kingdom; 3 National Hospital Organization Ryukyu Hospital Kin-Town Kunigami-gun Okinawa Japan; 4 Research Center for Child Mental Development University of Fukui Fukui Japan; 5 Department of Pediatrics and Child Health Kurume University School of Medicine Kurume Japan; 6 Institute of Economic Research Hitotsubashi University Tokyo Japan; 7 United Graduate School of Child Development Osaka University Kanazawa University, Hamamatsu University School of Medicine, Chiba University and University of Fukui, Osaka University Yamadaoka Suita, Osaka Japan; 8 Department of Child and Adolescent Psychological Medicine University of Fukui Hospital Fukui Japan

**Keywords:** ADHD, parent training, Japan, New Forest Parent Programme, parent stress management

## Abstract

**Background:**

Attention-deficit/hyperactivity disorder (ADHD) is a common neurodevelopmental disorder associated with numerous functional deficits and poor long-term outcomes. Internationally, behavioral interventions are recommended as part of a multimodal treatment approach for children with ADHD. Currently, in Japan, there are limited interventions available to target ADHD. Well Parent Japan (WPJ), a new hybrid parent-training program, provides a culturally acceptable and effective way to help support Japanese children with ADHD and their parents.

**Objective:**

This pragmatic multicenter randomized controlled trial aims to provide preliminary evidence about the effectiveness and cost-effectiveness of WPJ evaluated against treatment as usual (TAU) within routine Japanese mental health services.

**Methods:**

Mothers of children (aged 6-12 years) diagnosed with ADHD were recruited from child and adolescent mental health care services at three hospital sites across Japan (Fukui, Fukuoka, and Okinawa). The mothers were randomized to receive immediate treatment or TAU. The effectiveness and cost-effectiveness of WPJ over TAU at the end of the intervention and at 3-month follow-up will be evaluated. The primary outcome is maternal parent domain stress in the parenting role. The following secondary outcomes will be explored: child behavior, including severity of ADHD symptoms; parenting practices; emotional well-being; and the parent-child relationship and maternal child domain parenting stress. Data analysis will follow intention-to-treat principles with treatment effects quantified through analysis of covariance using multilevel modeling. An incremental cost-effectiveness ratio will be used to analyze the cost-effectiveness of the WPJ intervention.

**Results:**

Study funding was secured through a proof-of-concept grant in July 2018. Approval by the institutional review board for the data collection sites was obtained between 2017 and 2019. Data collection began in August 2019 and was completed in April 2022. Participant recruitment (N=124) was completed in May 2021. Effectiveness and cost-effectiveness analyses are expected to be completed by July 2022 and December 2022, respectively. These timelines are subject to change owing to the COVID-19 pandemic.

**Conclusions:**

This is the first multisite pragmatic trial of WPJ based on the recruitment of children referred directly to routine clinical services in Japan. This multisite randomized trial tests the effectiveness of WPJ in children and families by comparing WPJ directly with the usual clinical care offered for children diagnosed with ADHD in Japan. We also seek to assess and compare the cost-effectiveness of WPJ with TAU in Japan.

**Trial Registration:**

International Standard Randomised Controlled Trial Number ISRCTN66978270; https://www.isrctn.com/ISRCTN66978270

**International Registered Report Identifier (IRRID):**

DERR1-10.2196/32693

## Introduction

### Background

Attention-deficit/hyperactivity disorder (ADHD) is a common neurodevelopmental disorder with an estimated worldwide prevalence of approximately 5% [1]. In Japan, the prevalence estimates range from 2.5% [2] to 10% [3]. The disorder is associated with numerous functional impairments and poor long-term outcomes [4,5] and negatively affects parents’ emotional well-being, parenting practices, and the parent-child relationship [6]. Effective interventions for children with ADHD and their families are therefore a high priority [7].

Current clinical guidelines recommend a multimodal treatment approach for ADHD that incorporates both pharmacological and nonpharmacological treatment options [8-10]. Parenting interventions are an example of a recommended nonpharmacological treatment option with proven efficacy in numerous clinical trials [11,12]. Recommended interventions generally provide parents with behavioral strategies aimed at increasing the frequency of appropriate behavior while reducing the frequency of unwanted behavior in children. The needs of families of children with ADHD have received less attention, with only a few studies directly targeting parental well-being [13,14].

### Behavioral Interventions for ADHD in Japan

Parent-training interventions are becoming more widely accepted in Japan with increased implementation, especially in community settings. Although efforts have been made to develop programs to address the needs of families of children with a range of neurodevelopmental disorders [15], the availability of interventions specifically designed for ADHD remains limited.

To our knowledge, none of the behavior programs currently being implemented in Japan includes specific elements to address parental coping or emotional well-being. In addition, nothing is known about the cost-effectiveness of behavioral interventions for ADHD in Japan.

### Development of Well Parent Japan

For Japanese families, the 8-session group-based New Forest Parenting Programme (NFPP) has been front-loaded with 5 additional sessions designed to increase mother’s understanding of ADHD and to address their psychological well-being and readiness to complete the NFPP program. These sessions were added on the recommendation of participants in our pilot and proof-of-concept studies [16] to provide additional support for mothers. Culturally, Japanese mothers are held responsible for their child’s behavior and struggle to request support for themselves and their children. These 5 sessions included auxiliary psychoeducation about ADHD and culturally tailored stress management training, cognitive restructuring, strategies for effective communication, and problem-solving skills, adapted from the 9-week parent stress management program [14].

The NFPP has been specifically developed for the treatment of ADHD. In addition to behavioral strategies to manage oppositional and defiant behavior, it includes games and activities targeting purported neuropsychological deficits underlying some of the symptoms of ADHD [17], designed to enhance the child’s cognition and self-regulation [18]. Numerous randomized controlled trials (RCTs) have shown that the receipt of NFPP is associated with a reduction in ADHD symptoms and improvements in parental well-being [18-21]. With the support of the program developers, the NFPP sessions were specifically adapted for use with Japanese families [16].

A recent pilot RCT of Well Parent Japan (WPJ) [22] demonstrated that participation in the program was associated with several positive outcomes compared with the wait-list control group; that is, significant reductions in parenting stress, higher levels of parenting self-esteem and use of more effective parenting strategies, reduced child aggression, internalizing problems, and a trend toward reduced inattention. Mothers who participated in WPJ also responded less negatively toward their children. WPJ appears to be an efficacious psychosocial intervention for ADHD in Japan, with the group format and the session content well tolerated.

### Objectives

This study aims to extend the results from the pilot RCT [22] to a larger multicenter pragmatic trial to assess the effectiveness and cost-effectiveness of WPJ compared with treatment as usual (TAU) in child and adolescent mental health care services in Japanese hospitals. WPJ will be compared against TAU at the end of the intervention and again after 3 months (short-term follow-up).

The primary objective is to compare the effectiveness of WPJ against TAU on maternal parent domain parenting stress, that is perceived stress in the parent-child dyad arising from characteristics of the parent, measured with the Parent Stress Index [23,24]. Parent domain stress was selected as the primary outcome measure given WPJ’s strong focus on the emotional well-being of participating mothers. Secondary objectives include comparison of the effectiveness of WPJ against TAU in improving child behavior, parental well-being, and parenting practices and to explore the cost-effectiveness of WPJ.

## Methods

### Study Design

**Figure 1:**
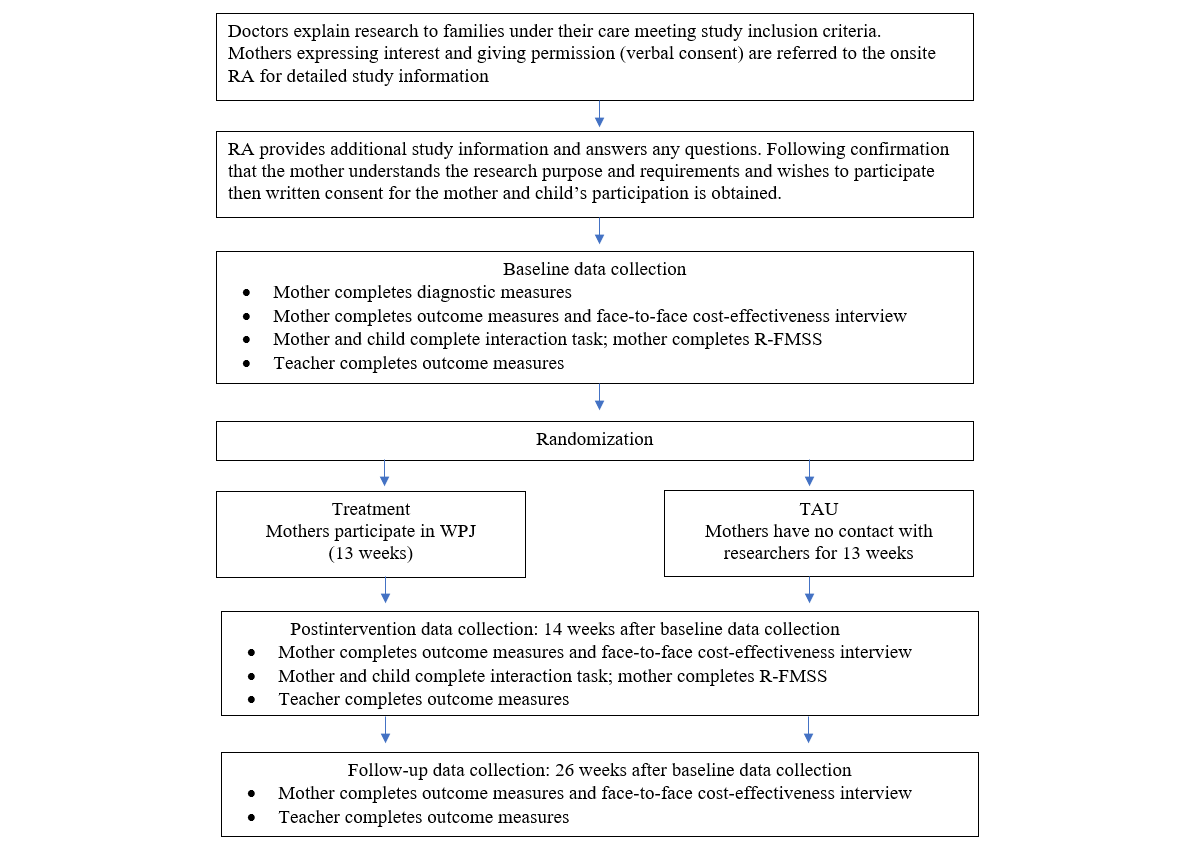
Study design and participant flow. R-FMSS: revised Five-Minute Speech Sample; RA: research administrator; TAU: treatment as usual; WPJ: Well Parent Japan.

A multisite pragmatic RCT comparing the effectiveness and cost-effectiveness of WPJ (treatment arm) with TAU (control arm). Both arms will be tested at baseline, immediately after the intervention arm completes WPJ at week 14, and at 3 months follow-up; that is, week 26. The study is being carried out through Japanese child and adolescent mental health care services at three different hospital sites in Japan (Fukui, Fukuoka, and Okinawa). Table 1 lists the schedule of parent self-report, teacher, and objective assessments at each time point, whereas Figure 1 summarizes the study design and flow of participants through the trial.

**Table 1:** Standard Protocol Items: Recommendations for Interventional Trials schedule.

Time point			Screening	Baseline	Intervention	Posttreatment (week 14)	3-month follow-up (week 26)
**Enrollment**							
	Informed consent		✓				
	Informed assent-child			✓			
**Randomization**							
	Well Parent Japan				✓		
	TAU^a^						
**Assessments**							
	**Diagnostic measures**						
		AQ^b^-child		✓			
		CAARS^c^-adult		✓			
	**Child behavior rating scales—Mother**						
		SNAP^d^ parent		✓		✓	✓
		Vanderbilt-Parent		✓		✓	✓
		Impairment Rating Scale		✓		✓	✓
	**Child behavior rating scales—Teacher**						
		SNAP teacher		✓		✓	✓
		Vanderbilt-teacher		✓		✓	✓
	**Mother self-report scales**						
		PSI^e^		✓		✓	✓
		Parenting scale		✓		✓	✓
		PSOC^f^		✓		✓	✓
		PLOC^g^		✓		✓	✓
		Family strain		✓		✓	✓
		BDI-2^h^		✓		✓	✓
	**Mother-child interaction measures**						
		SCIFF^i^ and SCIPD^j^		✓		✓	
		R-FMSS^k^		✓		✓	
	**Cost-effectiveness interview**						
		JHEC^l^		✓		✓	✓

^a^TAU: treatment as usual.

^b^AQ: Autism Quotient.

^c^CAARS: Conners Adult ADHD Rating Scales.

^d^SNAP: Swanson, Nolan, and Pelham Scale.

^e^PSI: Parenting Stress Index.

^f^PSOC: Parenting Sense of Competence.

^g^PLOC: Parental Locus of Control.

^h^BDI-2: Beck Depression Inventory-2.

^i^SCIFF: System for Coding Interactions and Family Functioning.

^j^SCIPD: System for Coding Interactions in Parent-Child Dyads.

^k^R-FMSS: Revised Five-Minute Speech Sample.

^l^JHEC: Japanese Health Economic Cost.

### Trial Registration

The study was retrospectively registered with the International Standard Randomised Controlled Trial Number after the first of 3 waves of participant recruitment (trial registration number: ISRCTN66978270). Registration delay was an unintended consequence of ill health of one of the principal investigators, which disrupted study preparation. This oversight was identified after the first wave of participant recruitment and the trial was subsequently registered. Ethical approval for the trial, as described in the protocol, was obtained at all intervention sites before participant recruitment.

### Sample Size Calculation

Parent domain parenting stress score at week 14 is the primary outcome measure and informed our sample size calculation. On the basis of the results of our pilot RCT [22], to detect a 0.5 standardized effect size at week 14 using 80% power at a 2-tailed .005 significance level, assuming the correlation between the baseline and follow-up measures is 0.35, 112 participants were required. After adjusting for a 15% attrition rate, the target sample size was inflated to 132.

### Sample Selection

#### Overview

Mothers of children with a clinical diagnosis of ADHD [1] were identified by physicians working in hospitals or community clinics linked to the 3 research sites.

#### Inclusion Criteria

Mothers fluent in Japanese (reading and writing), parenting a child aged 6-12 years diagnosed with ADHD and attending elementary school, and for whom participation in a group-based behavioral intervention for the mother is not contraindicated were eligible for inclusion in the study. In addition, mothers of children diagnosed with ADHD and autism spectrum disorder were eligible to participate. Referring doctors were asked to exercise clinical judgment regarding mothers’ ability to understand the program content and their suitability to participate in a group program.

#### Exclusion Criteria

Limited pragmatic speech or a functional intellectual disability in the child would exclude a family from participating in the study. Current or recent (ie, within 2 months of the starting date of WPJ) participation in another parenting program would also exclude participation. Mothers of children receiving medication for the management of their ADHD symptoms were asked to keep their child’s medication constant throughout the study. Medication status changes will be recorded but will not result in the family being removed from the trial.

### Consent and Randomization

Physicians at the 3 hospital study sites confirmed the eligibility of the mothers of their patients with ADHD to participate in the study. They briefly explained WPJ and the requirements for research participation. Interested mothers were referred to the research site administrator for a detailed verbal and written explanation of the study procedures, including randomization to WPJ or TAU and the need to access the child’s medical records. Those who agreed to participate provided written consent for their own participation and their child’s participation. Written consent from teachers was obtained by mail. Children’s informed consent was obtained at the first laboratory assessment session.

Baseline outcome measures were obtained from mothers and teachers, including the Revised Five-Minute Speech Sample (R-FMSS) and the parent-child interaction task, before randomization to the study arm (Table 1 provides the full list of baseline measures). Teachers remained blind to family treatment group allocation. When sufficient participants were recruited at a site (a minimum of 12), they were randomized to the study arm, that is, block randomization at each site, using a computer random number generator operated by DD.

### Confidentiality

All participants were assigned an ID number at each site. Raw data were stored in locked storage at the collection sites. Only anonymized data were shared with researchers at the primary research site (Okinawa Institute of Science and Technology [OIST]) for database entry and analyses.

### Intervention

#### Arm 1: WPJ

Participants received a 13-session group-based intervention for parents of children with ADHD. This includes an orientation to the intervention, psychoeducation about ADHD, 4 sessions devoted to enhancing mothers’ psychological functioning adapted from the Parent Stress Management for ADHD program [14], followed by 8 sessions of behavior management based on the core components of the NFPP [18-22]. A summary of the session contents is presented in Table 2. Mothers and teachers completed outcome measures, including parent domain parenting stress, before randomization, after the intervention, and again after 3 months. Table 1 provides a complete list of the measures completed at each time point.

The 2-hour WPJ sessions are run by 2 group leaders at each site. Participants who are unable to attend a group session are invited to a 30-60 catch-up session before the next group session. The catch-up sessions are limited to a maximum of 2 sessions per participant. To maintain treatment fidelity, the groups are run according to the WPJ leader’s manual. At the end of each session, the leaders complete a checklist of the topics covered. Missed information is presented at the beginning of the next group session. Approximately 20% of the sessions delivered at each site will be randomly selected to confirm fidelity. These recordings will be reviewed independently against a checklist of the key and minor points to be covered in each session. The percentage of inclusion of major and minor points will be calculated and reported.

Group leaders receive supervision from SS, a certified trainer in both interventions that comprise WPJ. Supervision sessions for the first wave of groups lasted 60-120 minutes, depending on the experience and requirements of therapists. The frequency and duration of supervision were reduced to 60 minutes biweekly thereafter. Supervision of the first 2 treatment groups was conducted separately by site. Group supervision, that is, 3 sites together, was planned from the beginning of the third group. All supervision took place via electronic platforms.

**Table 2:** Well Parent Japan session content.

Session	Content	Session function
1	Orientation to the program: What is ADHD^a^, part 1	Psychoeducation
2	Stress management, relaxation training	Mothers emotional health
3	Cognitive restructuring	Mothers emotional health
4	Problem solving	Mothers emotional health
5	Communication skills	Mothers emotional health
6	What is ADHD, part 2; Recruiting attention, positive communication, praise	Psychoeducation; New Forest Parenting Programme
7	Zone of proximal development, choices, clear messages, countdowns, using a timer	New Forest Parenting Programme
8	Review of session 7 skills, use of play	New Forest Parenting Programme
9	House rules, routine, boundaries, reward, and punishment	New Forest Parenting Programme
10	Review of session 9, review of ADHD symptoms	New Forest Parenting Programme
11	Temper tantrums (time out, quiet time), anticipating, avoiding conflict	New Forest Parenting Programme
12	Emotion and language, child relaxation	New Forest Parenting Programme
13	Social stories, mindfulness, wrap up session	New Forest Parenting Programme

^a^ADHD: attention-deficit/hyperactivity disorder.

#### Arm 2: TAU

TAU within the context of this trial may consist of many forms of intervention, including the following: medical and psychological examinations, pharmacological treatment, ADHD psychoeducation and parenting guidance, psychological counseling, play therapy for the child, group-based cognitive training for the child, telephone counseling for parents and teachers, and parent and teacher conferences or anything deemed clinically necessary. Mothers in the TAU arm had no contact with the research team from randomization until the treatment group (arm 1) completed the 13-week WPJ program. At this time, they attended a second laboratory assessment session with their child and completed all questionnaires. They completed the questionnaires again 3 months later. Site principal investigators will review and report the treatment options provided to all participating families (WPJ and TAU groups) through the referring hospitals during the trial period. This information will be included in the primary trial paper. The treatment group parents are given a 1000-yen (US $8.5) voucher per intervention session attended to help cover travel costs. All parents received a 2000-yen (US $17) voucher for the prelaboratory and postlaboratory assessment sessions and a further 1000-yen (US $8.5) voucher for the third cost-effectiveness interview. Teachers were given a 1000-yen (US $8.5) voucher each time they filled out the study questionnaires.

### Measurements

#### Diagnostic Measures

At baseline, autistic traits in the children were measured using the Autism-Spectrum Quotient [25]. This measure has excellent psychometric properties [26] and has been translated into Japanese and evaluated in both children [27] and adults [28]. The participating mothers’ ADHD traits are assessed using the Conners Adult ADHD Rating Scale [29] to better quantify the sample. The Japanese version of the scale has good psychometric properties [30].

#### Outcome Measures

##### Overview

Some of the measures (ie, the Vanderbilt Assessment Scale-Parent and Teacher, Parenting Sense of Competence Scale, Parental Locus of Control Scale, Family Strain Index, and Impairment Rating Scale) were translated into Japanese by the first author (SS) and independently back-translated by 2 bilingual US trained counseling psychologists.

The primary outcome measure was the mother’s reported parent domain parenting stress. This was assessed using the 78-item Japanese language version of the Parenting Stress Index [23]. The Parenting Stress Index assesses perceived stress in the parent-child dyad and yields the parent domain (sources of stress related to parent characteristics) and child domain stress (sources of stress related to child characteristics) scores and total stress score. The Japanese version of this measure has good psychometric properties [24].

Secondary outcome measures include parent and teacher ratings of the child’s behavior and mothers’ self-reports of their parenting style, competence, locus of control, family strain, mood, and objective measures of parent-child relationship quality. The measures are listed in Table 3.

All outcome measures, except for relationship quality, are administered at baseline, post treatment (week 14), and at the 3-month follow-up (week 26). Relationship quality measures are assessed at baseline and week 14 only. Teachers, but not parents, are blinded (probably) to parents’ group membership; that is, parents are asked not to discuss their group assignment with the child’s teacher.

**Table 3:** Primary and secondary outcomes, measures, and raters.

Outcomes			Measures	Raters
**Primary**				
	Parent domain stress		Parent Stress Index	Mother
**Secondary**				
	**Child behavior**			
		ADHD^a^ symptoms	Swanson, Nolan, and Pelham Scale	Mother and teacher
		Impairment	Impairment Rating Scale	Mother
		Other	Vanderbilt Assessment Scale	Mother and teacher
	**Parenting practices or attitudes**			
		Management practices	Parenting Scale	Mother
		Locus of control	Parental Locus of Control	Mother
		Sense of competence	Parenting Sense of Competence	Mother
	**Emotional well-being**			
		Depression	Beck Depression Inventory-2	Mother
		Caregiver strain	Family Strain Index	Mother
		Child domain stress	Parent Stress Index	Mother
	**Mother-child relationship**			
		Parent-child interactions	Pasta making task; SCIFF^b^, SCIPD^c^	Independent coders
		Expressed emotion	Revised 5-minute speech sample	Independent coders

^a^ADHD: attention-deficit/hyperactivity disorder.

^b^SCIFF: System for Coding Interactions and Family Functioning.

^c^SCIPD: System for Coding Interactions in Parent-Child Dyads.

##### Parent and Teacher Ratings of Child Outcomes

The 26-item Swanson, Nolan, and Pelham Scale [31] is used to measure the child’s ADHD and oppositional defiant disorder symptoms. The Japanese translation has excellent psychometric properties [32]. The Parent and Teacher performance scales from the Vanderbilt Assessment Scale [33] are used to assess the child’s behavioral and academic performance at school. The 8-item Impairment Rating Scale [34] is used to assess parents’ perceptions of child impairment across a range of domains. This measure demonstrates acceptable psychometric properties including good temporal stability [34].

##### Parent Self-report Outcomes

Parenting practices are assessed using the 30-item Parenting Scale [35]. The Japanese version has a 2-factor solution and shows good internal consistency for overreactivity and low to moderate consistency for laxness in control and clinical samples [36]. The 17-item Parenting Sense of Competence Scale [37,38] is used to assess parenting competence or efficacy. The Japanese translation demonstrated adequate internal consistency in a normative Japanese sample [39]. The 47-item Parental Locus of Control Scale [40] is used to assess parents’ perceived locus of control in child-rearing situations. The internal consistency of this measure in our pilot RCT was good [22]. The Family Strain Index [41] is used to assess the effects of ADHD on families. Items 3 and 5 of this 6-item index have been revised to make the descriptions more culturally appropriate for Japanese parents. The second edition of the Beck Depression Inventory [42] is used to assess the mothers’ levels of depression. The Japanese translation is psychometrically robust and can be used to measure depressive symptoms in Japanese populations [43].

##### Parent-Child Relationship Outcomes

The quality of mother-child interactions is evaluated through direct observation of behavior during a cooperative task. Each mother-child pair works together for 15 minutes to make pasta using the ingredients and equipment supplied. The interaction is video recorded for later coding using selected parent and child codes from the System for Coding Interactions and Family Functioning [44] and the System for Coding Interactions in Parent-Child Dyads [22,45]. Maternal expressed emotion is measured using the R-FMSS [46]. Parents speak for 5 minutes, without interruption, about their child and their relationship. The R-FMSS has demonstrated excellent psychometric properties [46]. Trained raters, at OIST, blind to group membership and time point, will code the parent-child interactions and the R-FMSS.

#### Economic Evaluation Measures

Health economic costs are measured using a study-specific measure (Japanese Health Economic Costs [JHEC]) to collect all available information to estimate the child’s service utilization costs (medical treatment, education, nursing, and rehabilitation) and parents’ opportunity costs for their child’s care. The JHEC is administered at baseline, after the intervention, and at 3-month follow-up. The JHEC was developed with reference to the Client Service Receipt Inventory [47].

### Data Management Plan

All the participants are assigned an ID number at the trial site when they consent to participate in the study. This number is used on all questionnaires. Each site will maintain a list allowing them to link names and ID numbers.

Data collection at each clinical site is supported by a dedicated research assistant with oversight and support from the trial team at OIST. Anonymized questionnaire data will be scanned, password-protected, and saved in a shared Dropbox with the researchers at OIST. Video and audio recordings will be password-protected and saved in the same shared Dropbox. The OIST trial team will transfer the data from the shared Dropbox folder to secure electronic storage at OIST as soon as it is received. The OIST team will not have access to the data linking lists from any site. Data entry into a password-protected electronic database will be carried out by the trial staff at OIST.

Raw data (possibly containing names) will be maintained in secure storage at each site for 10 years after study completion. Access to these data is limited to the site principal investigator and dedicated research assistant. Once all data analyses are complete and manuscripts are prepared, OIST-maintained electronic password-locked files will be wiped. OIST will retain the anonymous password-protected data for 10 years after manuscript publication. GT will be responsible for the supervision of stored data.

Data analysis will be carried out by the trial statistician using an anonymized electronic database (see the Statistical Analysis section). Although there are no plans to make the data publicly available, requests for access to the data will be considered on a case-by-case basis, subject to Japanese law.

### Safety Considerations

Therapists are asked to record and report any potential adverse events using a standard template, and the importance of adverse events is highlighted during supervision sessions. Any adverse events are required to be reported to the approving ethics committees. In addition, all adverse events will be reported to the independent data and ethics monitor and will be evaluated, recorded, and discussed in the final paper.

### Statistical Analysis

#### Effectiveness Analyses

The analysis will be conducted on an intention-to-treat basis. Data will be explored first, and all variables will be summarized by treatment arm across measurement time, with mean (SD) presented for normally distributed measures, median (IQR) for skewed data, and frequency (%) for each observed level of categorical variables. Treatment effect estimates and their precision on the primary and secondary outcomes will be quantified through analysis of covariance modeling by means of multilevel modeling with baseline measure, arm, time, and interaction of arm × time included as covariates and participant as a level 2 analytical unit. As participants are recruited from different locations, the site will be included as a higher-level analytical unit if exploratory data analysis shows greater variability at the site level. Skewed outcome variables will be transformed for parameter modeling. Missing value information will be explored first to inform the missing value imputation by means of a multiple imputation procedure for multilevel data under a missing-at-random assumption. The results from the observed data will be examined to check the robustness of the treatment effect estimates sensitive to missingness. Stata statistical software (version 17; StataCorp) [48] will be used for data analysis. A statistical analysis plan, including all the analytical details, will be conducted before the data are locked for final analysis.

An independent data and ethics monitor is responsible for reviewing the trial progress and data analysis once data collection is complete. The monitor is independent of both the study team and sponsor OIST.

#### Cost and Cost-effectiveness Analyses

The health economic evaluation will focus on the three following key areas:

1. Costs of the intervention: an average cost per intervention will be determined. This analysis will include staffing costs (hourly rate × time) from study time sheets, capital costs (eg, rooms and overheads), and consumables. Parent time costs will not be included in this analysis.
2. Cost-effectiveness: an incremental cost-effectiveness ratio will be used to analyze the cost-effectiveness of the WPJ intervention. The incremental cost-effectiveness ratio will be able to identify differences in costs such as health care and educational utilization between the intervention and TAU arms of the trial divided by the treatment effect, the proportion of participants meeting the criteria for positive treatment response (changes in maternal stress as measured by the Parenting Stress Index [24]) and child ADHD symptoms as measured by the index of the parent-completed SNAP [32]. The JHEC and medical receipts provided by the participants will be used to track the familial and societal costs of health and educational service resource use in both study arms. This study will seek to use a number of routine health service costs, where possible, using data from the Japanese Medical Fee Points system [49,50]. This analysis will be completed with and without family borne costs. Any loss of earnings will be calculated using the average wage rate.
3. Impact of intervention on service use: this study will measure the retrospective change in resource use across both trial arms from 3 months before each assessment point. This analysis will use data from the JHEC and medical receipts provided by the participants.

### Data Sharing Statement

Although there are no plans to make the data publicly available, requests for access to the data will be considered on a case-by-case basis, subject to Japanese law.

### Ethics Statement

The study has been approved by the OIST Graduate School Human Subject Research Review Committee (reference number: HSR-2019-014) following receipt of approval at each study site: University of Fukui Hospital (reference number: 20170085), Kurume University Hospital (reference number: 19052), and National Hospital Organization Ryukyu Hospital (reference number: 31-5).

## Results

The Training and Nurturing Support for Mothers (TRANSFORM) study began in July 2019 with therapist training, and a practice intervention group was run at each site between July 2019 and October 2019. Trial recruitment began in July 2019 and was completed in May 2021. The timeline of the study has been extended because of the limitations imposed by the COVID-19 pandemic. This included a 5-month postponement of the second treatment group (all 3 sites) and suspension of the third treatment group (2 sites). At the time of protocol submission, the suspension remained in place at 1 site. These suspensions will be reported in the final paper.

## Discussion

### Principal Findings

This trial addresses the need to investigate the effect of nonpharmacological treatments for children with ADHD in everyday clinical practice in Japan. This trial combines 2 evidence-based interventions for families of children with ADHD within a hybrid program that has been developed and piloted to provide a culturally adapted and appropriately delivered behavioral intervention for families of children with ADHD in Japan. Combining support for families of children with ADHD with behavioral strategies that aim to target and alter the developmental trajectory of ADHD has implications for researchers and clinicians beyond Japan. The results will be reported at national and international conferences, published in peer-reviewed journals, disseminated to Japanese health care professionals, and communicated digitally to Japanese and international ADHD advocacy groups.

This is the first trial of WPJ based on the recruitment of children referred directly to routine clinical services in Japan. It is also the first pragmatic trial that combines the NFPP with specific and direct intervention support for parents of children with ADHD. In this sense, this multisite randomized trial tests the effectiveness of WPJ with children and families in a clinical setting for which the intervention is ultimately intended with very few exclusion criteria. It also compares WPJ directly with the usual clinical care offered for children diagnosed with ADHD in Japan. We also seek to assess and compare the cost-effectiveness of WPJ with that of TAU in Japan.

The stigma associated with ADHD in Japan and unfamiliarity with group-based interventions for ADHD posed challenges to recruitment. The COVID-19 pandemic continues to affect the trial, with local and national lockdowns and restrictions affecting recruitment and disrupting the process of delivering the intervention. The decision to provide supervision via web-based platforms ensured that this aspect of the study has not been disrupted by the COVID-19 pandemic. In principle, the group delivery model is a challenge for parents of children with ADHD, who often perform better with individual therapy, where a bespoke response can address the heterogeneity of the child’s ADHD. However, in the proof-of-concept study, a group model was requested by Japanese mothers [16]. Culturally, it was felt that an individual approach might not be appropriate, which was supported by the pilot RCT that demonstrated considerable levels of efficacy against a wait-list control group [22] using a group-based mode of delivery. Establishing the cost-effectiveness of the intervention has also been challenging, as the academic discipline of health economics is not well-established in Japan. In addition, the process for calculating society and family borne costs, such as health care use in Japan, is complex, where health care costs are based on a point system that does not easily lend itself to economic evaluation. The COVID-19 pandemic has likely influenced family health service use, with families limiting their use of, and visits to, health care providers. We assume similar effects across the 2 study arms.

### Limitations

In recruiting through hospital clinics, the study does not include self-referred families in the community, possibly reducing the generalizability of the findings. Having clinic doctors (in Japan, the diagnosis of ADHD is limited to medical doctors; thus, referrals to the research were made by those responsible for the diagnosis of ADHD in the children) identify eligible families may have resulted in the referral of those dealing with more severe or comorbid ADHD, or mothers deemed most in need or most likely to benefit from participation in WPJ. Nonetheless, this is consistent with the aim of the research; that is, to evaluate WPJ within the Japanese child and adolescent mental health care system with a view toward future implementation of the intervention within this system. As a pragmatic trial, study site differences will be evaluated; however, we do not control for the effects of group leader skills on treatment outcomes. All group leaders are provided with the same training and are supervised by SS, which should help minimize differences in program delivery. The registration of the protocol was retrospective, and participant recruitment is now complete. No changes were made in the study protocol.

## Abbreviations

ADHD: attention-deficit/hyperactivity disorder
JHEC: Japanese Health Economic Costs
NFPP: New Forest Parenting Programme
OIST: Okinawa Institute of Science and Technology
R-FMSS: revised Five-Minute Speech Sample
RCT: randomized controlled trial
TAU: treatment as usual
WPJ: Well Parent Japan
